# Differences in Expert Perspectives on AI Training in Medical Education: Secondary Analysis of a Multinational Delphi Study

**DOI:** 10.2196/72186

**Published:** 2025-05-09

**Authors:** Qi Chwen Ong, Chin-Siang Ang, Nai Ming Lai, Rifat Atun, Josip Car

**Affiliations:** 1 School of Public Health, Imperial College London, White City Campus, 90 Wood Lane, London, W12 0BZ, United Kingdom, 44 20-7589-511; 2 Lee Kong Chian School of Medicine, Nanyang Technological University, Singapore, Singapore; 3 WHO Collaborating Centre for Digital Health and Health Education, Nanyang Technological University, Singapore, Singapore; 4 School of Medicine, Faculty of Health and Medical Sciences, Taylor's University, Subang Jaya, Malaysia; 5 Department of Global Health and Population, Harvard TH Chan School of Public Health, Harvard University, Cambridge, MA, United States; 6 Department of Global Health and Social Medicine, Harvard Medical School, Harvard University, Cambridge, MA, United States; 7 School of Life Course and Population Sciences, King's College London, London, United Kingdom

**Keywords:** artificial intelligence, medical education, competencies, health professions education, Delphi study, global health education, AI

## Abstract

In this secondary analysis of a multinational Delphi study, experts from low- and middle-income countries were less likely than those from high-income countries to consider artificial intelligence (AI) learning outcomes mandatory in preregistration medical education, potentially reflecting underlying global inequalities in medical AI education and highlighting the need for adaptable AI competency frameworks.

## Introduction

Artificial intelligence’s (AI) rapid advances in health care have intensified calls for incorporating AI training into medical education [1]. However, few existing AI-related medical curricula are tailored to specific national contexts and lack global applicability [2,3]. Perspectives on the relevance and prioritization of AI training may vary between high-income countries (HICs) and low- and middle-income countries (LMICs), as do the development, evaluation, and implementation of health care AI technologies [4,5]. Understanding differences in AI training priorities is crucial for designing medical curricula addressing global and national health care needs. We examined differences in perspectives on the prioritization of AI learning outcomes (LOs) in preregistration medical education between experts from HICs and LMICs.

## Methods

### Overview

We conducted a secondary analysis using deidentified data from a multinational 2-round modified Delphi study on digital health competencies in medical education (DECODE) [6]. This study involved 211 experts from 79 countries and territories recruited through purposive and snowball sampling based on expertise in digital health, health informatics, clinical medicine, or medical education. Only participants who completed both rounds of the Delphi survey were included in this analysis. The detailed Delphi methodology and description of AI LOs were reported elsewhere [6].

Participants rated 19 proposed LOs under the competency domain “Artificial Intelligence in Healthcare” from the DECODE framework (Multimedia Appendix 1) as mandatory, elective, or supplementary. The outcome was the binary conversion of ratings on prioritization (mandatory vs discretionary [elective/supplementary]) of AI LOs. The main exposure variable was participants’ self-reported country of primary affiliation, classified as either HIC or LMIC based on the World Bank’s income classification [7]. Differences in participant characteristics were assessed using Fisher exact tests. To account for participant-level clustering in repeated measures data and varying correlations between AI LOs (ie, conceptual, technical, and ethical aspects), we used generalized estimating equations with an unstructured correlation matrix to examine the association between participants’ ratings of AI LOs and their country income group. Models were adjusted for participants’ clinical background, research role, and leadership position. Variables such as clinical role, hospital workplace, teaching role, and university workplace were excluded due to multicollinearity. All analyses were performed using R version 4.3.1 (R Project for Statistical Computing), with a 2-sided *P*<.05 considered statistically significant.

### Ethical Considerations

The original Delphi study and this secondary analysis were approved by the Nanyang Technological University Institutional Review Board (IRB-2021‐838). Additional informed consent was not required for this secondary analysis due to the use of deidentified data.

## Results

Of the 149 experts who participated in both rounds of the original Delphi study, 130 (87.2%) completed the AI LO section (n=59, 45.4% from HICs and n=71, 54.6% from LMICs). Significant differences between HIC and LMIC participants were observed in teaching roles (*P*=.01), clinical setting (hospital or private practice; *P*=.03), or institutional leadership positions (*P*<.001; Table 1). In the adjusted model, experts from LMICs were significantly less likely to rate AI LOs as mandatory in medical education (odds ratio 0.58, 95% CI 0.37‐0.91; *P*=.02) compared to their HIC counterparts (Table 2).

**Table 1.** Characteristics of Delphi experts by country income groups.

Characteristics	Experts (n=130), n (%)		*P* value
	HICs^a^ (n=59)	LMICs^b^ (n=71)	
Primary professional discipline			>.99
Clinical medicine	41 (69)	50 (70)	
Nonclinical medicine	18 (31)	21 (30)	
Teaching role			.01
Yes	43 (73)	64 (90)	
No	16 (27)	7 (10)	
Clinical role			.50
Yes	32 (54)	33 (46)	
No	27 (46)	38 (54)	
Research role			.40
Yes	40 (68)	53 (75)	
No	19 (32)	18 (25)	
Institutional leadership position^c^			<.001
Yes	11 (19)	34 (48)	
No	48 (81)	37 (52)	
Worked in university			.50
Yes	56 (95)	65 (92)	
No	3 (5)	6 (8)	
Worked in hospital/private practice			.03
Yes	35 (59)	28 (39)	
No	24 (41)	43 (61)	

a HIC: high-income country.

b LMIC: low- and middle-income countries.

c Defined as president (or vice president), chancellor, or rector of a university; dean or vice dean of a medical school or faculty; or chief medical officer or chief medical informatics officer of a health care institution.

**Table 2.** Inclusion of artificial intelligence learning outcomes in medical education by country income groups.

Learning outcome	Unadjusted OR^a^ (95% CI)	*P* value	Adjusted OR^b^ (95% CI)	*P* value
Mandatory inclusion of artificial intelligence learning outcomes				
Low- and middle-income countries	0.64 (0.43‐0.95)	.03^c^	0.58 (0.37‐0.91)	.02^c^
Clinical medicine background	—^d^	—	0.86 (0.56‐1.31)	.47
Research role	—	—	0.86 (0.58‐1.26)	.44
Institutional leadership	—	—	1.34 (0.86‐2.09)	.19

a OR: odds ratio.

b Adjusted for clinical medicine background, research role, and institutional leadership position.

c *P*<.05.

d Not applicable.

## Discussion

In this secondary analysis of data from 130 experts, those from LMICs appeared less likely to consider AI LOs as mandatory compared to their HIC counterparts, which may have implications for widening inequalities in medical AI expertise between HICs and LMICs. Our findings extend research on regional variation in attitudes toward AI in health care. One cross-sectional study revealed differing views on AI in practice between health profession students from the Global North and Global South [8], while another study reported lower confidence in clinical AI among physicians and medical students from lower middle– and low-income countries compared to those from upper middle– and high-income countries [9]. The congruence of our findings with these studies points to a potential divergence in AI perceptions across income settings, likely reflecting broader structural inequalities such as disparities in digital infrastructure, educational resources, institutional readiness, and exposure to AI technologies, rather than disagreement on the value of AI training.

Several limitations should be noted. Binary classification of countries may mask important intragroup differences. Imbalanced representation of participant roles may have introduced bias, and important demographic variables such as age, gender, and years of experience were not available. Expert migration, institutional policies, and exposure to AI may also have influenced expert responses. Future research should use qualitative methods to elucidate contextual determinants of expert opinions on AI training.

In conclusion, our findings indicate that a one-size-fits-all approach to AI training in medical education may not be appropriate. An adaptable, needs-based framework that considers socioeconomic, infrastructural, and health care disparities could better serve the diverse needs of medical students and health systems worldwide.

## Supplementary material

10.2196/72186 Multimedia Appendix 1 Ratings of artificial intelligence learning outcomes in 2-round modified Delphi survey.

## Abbreviations

AI: artificial intelligence
DECODE: digital health competencies in medical education
HIC: high-income country
LMIC: low- and middle-income countries
LO: learning outcome
