# Correction: Differences in Expert Perspectives on AI Training in Medical Education: Secondary Analysis of a Multinational Delphi Study

**DOI:** 10.2196/78155

**Published:** 2025-05-30

**Authors:** Qi Chwen Ong, Chin-Siang Ang, Nai Ming Lai, Rifat Atun, Josip Car

**Affiliations:** 1 School of Public Health, Imperial College London, White City Campus, 90 Wood Lane, London, W12 0BZ, United Kingdom, 44 20-7589-511; 2 Lee Kong Chian School of Medicine, Nanyang Technological University, Singapore, Singapore; 3 WHO Collaborating Centre for Digital Health and Health Education, Nanyang Technological University, Singapore, Singapore; 4 School of Medicine, Faculty of Health and Medical Sciences, Taylor's University, Subang Jaya, Malaysia; 5 Department of Global Health and Population, Harvard TH Chan School of Public Health, Harvard University, Cambridge, MA, United States; 6 Department of Global Health and Social Medicine, Harvard Medical School, Harvard University, Cambridge, MA, United States; 7 School of Life Course and Population Sciences, King's College London, London, United Kingdom

In “Differences in Expert Perspectives on AI Training in Medical Education: Secondary Analysis of a Multinational Delphi Study” (J Med Internet Res 2025;27:e72186) the authors noted one error.

The following affiliation was incorrectly stated as Affiliation 1, when it should have been Affiliation 7:

*School of Life Course and Population Sciences, King’s College London, London, United Kingdom*

This has been corrected, and the remaining affiliations moved up sequentially by 1 to compensate.

The correction will appear in the online version of the paper on the JMIR Publications website, together with the publication of this correction notice. Because this was made after submission to PubMed, PubMed Central, and other full-text repositories, the corrected article has also been resubmitted to those repositories.

