# Supplementary material for: Differences in Expert Perspectives on AI Training in Medical Education: Secondary Analysis of a Multinational Delphi Study
Source: J Med Internet Res. 2025 May 9;27:e72186. doi: 10.2196/72186 (PMC12083730; doi:10.2196/72186)
Supplement: Multimedia Appendix 1 [file jmir-v27-e72186-s001.docx]

**eMethods. Ratings of artificial intelligence learning outcomes in two-round modified Delphi survey**

In Round 1, Each participant was asked to rate the relevance of learning outcomes (LOs) within the competency “Artificial Intelligence in Healthcare” using a four-point Likert scale (strongly agree, agree, disagree, strongly disagree). LOs that achieved a pre-defined consensus threshold of ≥70% (i.e. combined percentage of participants who 'agreed' and 'strongly agreed') were considered relevant and retained for the next round. LOs that did not meet the threshold were dropped from the framework. In Round 2, experts rated the retained LOs on their recommendation for prioritisation in pre-registration medical education curricula, as either mandatory, elective, or supplementary. LOs that achieved a pre-defined consensus threshold of ≥70% (i.e. percentage of mandatory ratings) were included as mandatory LOs in the final DECODE framework, while those did not were included as discretionary LOs.
